# *Rhus coriaria* L. (Sumac) Demonstrates Oncostatic Activity in the Therapeutic and Preventive Model of Breast Carcinoma

**DOI:** 10.3390/ijms22010183

**Published:** 2020-12-26

**Authors:** Peter Kubatka, Martin Kello, Karol Kajo, Marek Samec, Alena Liskova, Karin Jasek, Lenka Koklesova, Tomas Kuruc, Marian Adamkov, Karel Smejkal, Emil Svajdlenka, Peter Solar, Martin Pec, Dietrich Büsselberg, Vladimira Sadlonova, Jan Mojzis

**Affiliations:** 1 Department of Medical Biology, Jessenius Faculty of Medicine, Comenius University in Bratislava, 036 01 Martin, Slovakia; martin.pec@uniba.sk; 2 Division of Oncology, Biomedical Center Martin, Comenius University in Bratislava, Jessenius Faculty of Medicine, 036 01 Martin, Slovakia; karin.jasek@uniba.sk; 3 Department of Pharmacology, Faculty of Medicine, P. J. Šafárik University, 040 11 Košice, Slovakia; kellomartin@yahoo.com (M.K.); tomas.kuruc@student.upjs.sk (T.K.); 4 Department of Pathology, St. Elisabeth Oncology Institute, 812 50 Bratislava, Slovakia; kkajo@ousa.sk; 5 Biomedical Research Center, Slovak Academy of Sciences, 845 05 Bratislava, Slovakia; 6 Department of Obstetrics and Gynecology, Jessenius Faculty of Medicine, Comenius University in Bratislava, 036 01 Martin, Slovakia; marek.samec@gmail.com (M.S.); alenka.liskova@gmail.com (A.L.); koklesova.lenka@gmail.com (L.K.); 7 Department of Histology and Embryology, Jessenius Faculty of Medicine, Comenius University in Bratislava, 036 01 Martin, Slovakia; marian.adamkov@uniba.sk; 8 Department of Natural Drugs, Faculty of Pharmacy, Masaryk University, 612 42 Brno, Czech Republic; smejkalk@pharm.muni.cz (K.S.); svajdlenkae@pharm.muni.cz (E.S.); 9 Department of Medical Biology, Faculty of Medicine, P. J. Šafárik University, 040 11 Kosice, Slovakia; peter.solar@upjs.sk; 10 Weill Cornell Medicine in Qatar, Qatar Foundation-Education City, 24144 Doha, Qatar; dib2015@qatar-med.cornell.edu; 11 Department of Microbiology and Immunology, Jessenius Faculty of Medicine, Comenius University in Bratislava, 036 01 Martin, Slovakia

**Keywords:** angiogenesis, apoptosis, breast cancer, cancer stem cells, cell proliferation, epigenetics, MCF-7 cells, MDA-MB-231 cells, mouse, rat, *Rhus coriaria*, sumac

## Abstract

Comprehensive scientific data provide evidence that isolated phytochemicals or whole plant foods may beneficially modify carcinogenesis. The aim of this study was to evaluate the oncostatic activities of *Rhus coriaria* L. (sumac) using animal models (rat and mouse), and cell lines of breast carcinoma. *R. coriaria* (as a powder) was administered through the diet at two concentrations (low dose: 0.1% (*w*/*w*) and high dose: 1 % (*w*/*w*)) for the duration of the experiment in a syngeneic 4T1 mouse and chemically-induced rat mammary carcinoma models. After autopsy, histopathological and molecular analyses of tumor samples in rodents were performed. Moreover, in vitro analyses using MCF-7 and MDA-MB-231 cells were conducted. The dominant metabolites present in tested *R. coriaria* methanolic extract were glycosides of gallic acid (possible gallotannins). In the mouse model, *R. coriaria* at a higher dose (1%) significantly decreased tumor volume by 27% when compared to controls. In addition, treated tumors showed significant dose-dependent decrease in mitotic activity index by 36.5% and 51% in comparison with the control group. In the chemoprevention study using rats, *R. coriaria* at a higher dose significantly reduced the tumor incidence by 20% and in lower dose non-significantly reduced tumor frequency by 29% when compared to controls. Evaluations of the mechanism of oncostatic action using valid clinical markers demonstrated several positive alterations in rat tumor cells after the treatment with *R. coriaria*. In this regard, histopathological analysis of treated tumor specimens showed robust dose-dependent decrease in the ratio of high-/low-grade carcinomas by 66% and 73% compared to controls. In treated rat carcinomas, we found significant caspase-3, Bax, and Bax/Bcl-2 expression increases; on the other side, a significant down-regulation of Bcl-2, Ki67, CD24, ALDH1, and EpCam expressions and MDA levels. When compared to control specimens, evaluation of epigenetic alterations in rat tumor cells in vivo showed significant dose-dependent decrease in lysine methylation status of H3K4m3 and H3K9m3 and dose-dependent increase in lysine acetylation in H4K16ac levels (H4K20m3 was not changed) in treated groups. However, only in lower dose of sumac were significant decreases in the expression of oncogenic miR210 and increase of tumor-suppressive miR145 (miR21, miR22, and miR155 were not changed) observed. Finally, only in lower sumac dose, significant decreases in methylation status of three out of five gene promoters–*ATM*, *PTEN*, and *TIMP3* (*PITX2* and *RASSF1* promoters were not changed). In vitro evaluations using methanolic extract of *R. coriaria* showed significant anticancer efficacy in MCF-7 and MDA-MB-231 cells (using Resazurin, cell cycle, annexin V/PI, caspase-3/7, Bcl-2, PARP, and mitochondrial membrane potential analyses). In conclusion, sumac demonstrated significant oncostatic activities in rodent models of breast carcinoma that were validated by mechanistic studies in vivo and in vitro.

## 1. Introduction

The global incidence and mortality burden of pre- and postmenopausal breast cancer continues to increase [1]. A hallmark of breast cancer tumor heterogeneity is irreversible impairment of cell function and homeostasis. Targeting key cellular processes, such as apoptosis, cell cycle, angiogenesis, and self-renewal of cancer stem cells, may effectively suppress the growth and spread of malignant cells [2]. In addition, several epigenetic molecular mechanisms, including DNA methylation patterns (i.e., hypermethylation of tumor-suppressor genes and hypomethylation of oncogenes), improper posttranslational histone chemical modifications, and the aberrant modulation in expression of microRNA, represent important drivers of carcinogenesis [3]. Spanning several decades, preclinical research has provided emerging evidence pertaining to the beneficial action of phytochemicals and whole plant foods on multiple cancer-related biological pathways [4,5,6,7,8,9]. Despite extensive preclinical research, only a limited number of clinical meta-analyses and epidemiological studies have described that long-term (several years) and regular (4–5 times a week) consumption of whole plant foods significantly decreases the risk of breast cancer [10,11,12,13].

The *Rhus coriaria* plant is widely consumed in the Mediterranean region and has been applied in traditional medicine for hundreds of years. *Rhus coriaria* L. (sumac) is a biotanical extracted from the *Rhus coriaria* plant, which displays high antioxidant activity [14]. Furthermore, sumac has gained notoriety due to its therapeutic potential in several diseases of civilization, including cancer [15]. Burgeoning evidence shows that this plant is rich in phytochemical compounds, such as flavonoids and hydrolysable tannins – mainly derivatives of gallic acid [16]. There are several papers documenting oncostatic activities of *R. coriaria* against cancer cell lines in vitro. Sumac suppressed tumor growth, metastasis, and neovascularity in the MDA-MB-231 breast cancer cell line by targeting the STAT3, NFκB, and nitric oxide signaling pathways [17]. The same research group described the induction of senescence and autophagic cell death by sumac in MDA-MB-231 cells through a mechanism involving p38 and ERK1/2 signaling activation [17]. Another study analyzed the anti-angiogenic potential of oleo gum resin extracts from *R. coriaria*, *Pistacia vera*, and *Pistacia khinjuk* against HUVEC cells [18]. Tube formation assay documented that extract of *R. coriaria* inhibited angiogenesis more effectively compared to both *Pistacia* extracts [18]. Apoptotic activity (increased Bax/Bcl-2 ratio) and anti-proliferative potential against MCF-7 breast cancer cells of silver nanoparticles synthesized from aqueous extract of sumac was described in another recent study [19]. Finally, in a study by Athamneh et al. (2017), *R. coriaria* up-regulated protein ubiquitination and proteasomal degradation, and triggered non-canonical Beclin-1-independent autophagy and apoptosis in HT-29 and Caco-2 human colon cancer cells [20].

The therapeutic and chemopreventive potential of *R. coriaria* has yet to be investigated in rodent breast cancer models. The purpose of this study was to assess the oncostatic efficacy of dietary administered *R. coriaria* using 4T1 syngeneic and chemically-induced mammary adenocarcinoma rodent models. The rationale for this study was established from our previous findings demonstrating significant oncostatic effects of natural mixtures of phytochemicals in in vivo and in vitro models of rodent breast cancer [21,22,23,24,25,26,27]. Allograft and chemoprevention models were used to define treatment potential (tumor volume) and cancer risk reduction (tumor incidence, frequency, and latency) after long-term administration of *R. coriaria*. With the aim to evaluate the oncostatic activities induced by *R. coriaria*, the validated clinical markers of apoptosis, proliferation, angiogenesis, oxidative damage, cancer stem cells, and cancer cell epigenetics (i.e., methylation status of gene promoters, markers of histone chemical modifications, and expression of selected miRNAs) were used. Moreover, the histopathological characteristics of cancer samples such as the mitotic index and tumor necrosis ratio in the therapeutic 4T1 model and the ratio of high/low grade carcinomas in the chemopreventive model were evaluated. Finally, the use of in vitro (MCF-7 and MDA-MB-231 cell lines) studies was aimed to improve mechanistic analyses (parameters of proliferation, cell cycle, and apoptosis in vitro) of the anticancer activities of sumac within the preclinical oncological research.

## 2. Results

### 2.1. Plant Secondary Metabolites in R. coriaria Methanolic Extract

**Figure 1:**
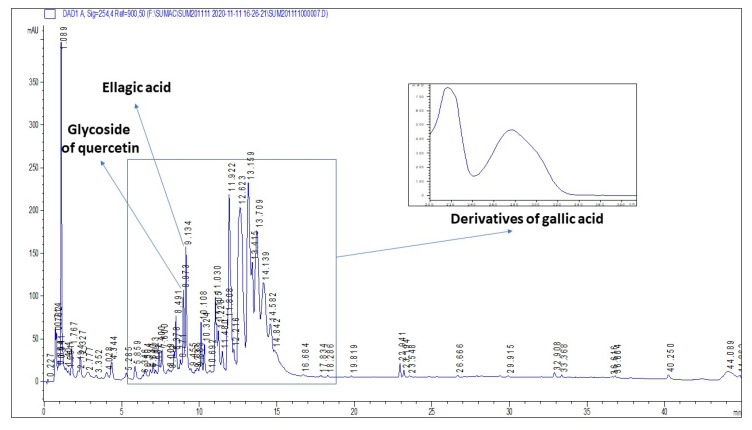
The representative chromatogram of methanolic extract of *R. coriaria*.

The methanolic extract of *R. coriaria* was prepared by a sonication of plant material, and consequently analyzed by HPLC. The HPLC-DAD showed the number of phenolic compounds spectrally matching the derivatives of gallic acid, usually assigned as gallotannins. We also identified the presence of ellagic acid. The retention times correspond to polar glycosidic compounds. Unfortunately, contrary to the literature reports, we were not able to detect large amounts of flavonoid glycosides, with the exception of a peak showing spectrum and retention corresponding to a derivative of quercetin. The representative chromatogram of the extract is shown by Figure 1.

### 2.2. 4T1 Therapeutic Model in Mice

**Figure 2:**
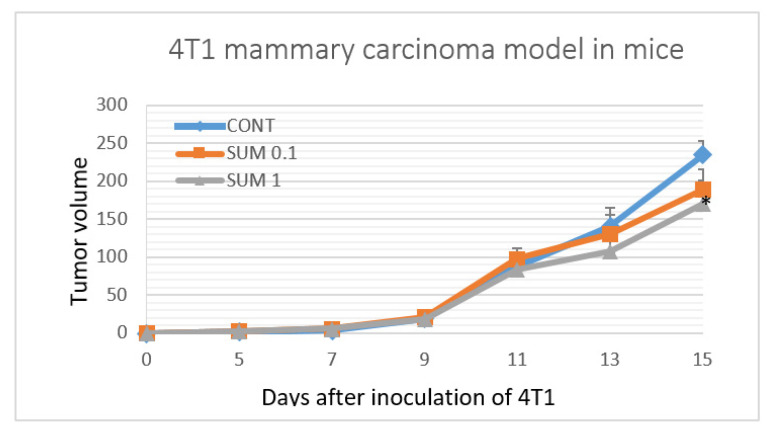
CONT—control group, SUM 0.1—group with administered sumac at a concentration of 1 g/kg in diet, SUM 1—group with administered sumac at a concentration of 10 g/kg in diet. The development of the volume of 4T1 mammary adenocarcinomas in mice allograft model during the experiment. Data are expressed as mean ± SEM. Significant differences: * *p* < 0.05 vs. CONT.

**Figure 3:**
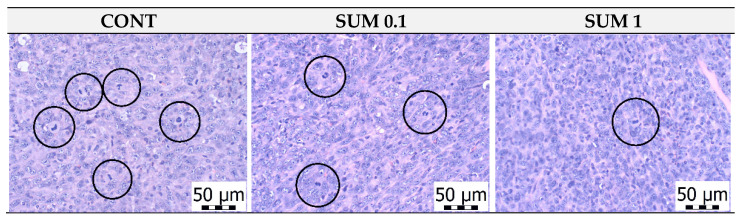
The mitotic activity index after treatment with *R. coriaria* in 4T1 tumors in Balb/c mice. The mitotic figures are highlighted in circles; H&E staining; magnification ×400.

Dietary administered of sumac significantly reduced the volume of 4T1 tumors in mice by 27% (*p* < 0.05) in the group receiving the higher dose compared to control animals (Figure 2). Histopathological analysis of 4T1 tumors demonstrated significant dose-dependent decrease in mitotic activity index in cancer cells after sumac treatment by 36.5% and 51% in comparison with the control group. (Table 1 and Figure 3). Assessing the parameter of necrosis/whole tumor area ratio, we did not observe any significant alterations when compared to control and treated mice.

### 2.3. Chemoprevention Model-Parameters of Rat Mammary Carcinogenesis and Histopathology of Tumors

*R. coriaria* significantly reduced the tumor incidence by 20 % (*p* < 0.05) in rats treated with the higher dose, when compared to controls (Table 2). Regarding other parameters of rodent mammary carcinogenesis, tumor frequency, latency, and volume were not significantly changed in treated groups compared to the control group (Table 2). However, an apparent decrease of tumor frequency (the key parameter of this model) by 29% was observed in the group receiving the lower sumac dose versus controls (with boundary significance). Mixed cribriform/papillary, alone cribriform, mixed papillary/cribriform, and cribriform/comedic carcinomas (dominant type is the first in order) were the most common mammary lesions observed in rats. We have observed only sporadic occurrence of mixed cribriform/papillary/comedo, papillary/cribriform/comedo, and tubular/papillary carcinomas and alone papillary carcinomas. Histopathological analysis of treated rat tumor specimens showed dose-dependent significant decreases in the high-/low-grade carcinomas ratios by 66% (*p* < 0.001) and 73% (*p* < 0.001) when compared to controls. 

### 2.4. Immunohistochemistry of Rat Tumors

**Figure 4:**
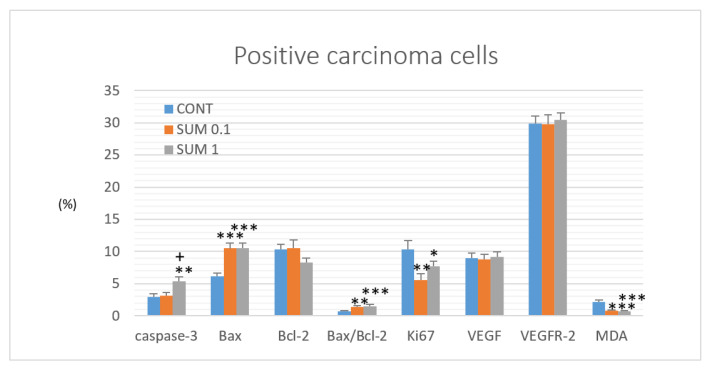
Immunohistochemical evaluation of cleaved caspase-3 (cytoplasmic), Bax, Bcl-2, Ki67, VEGFA, VEGFR-2, and MDA expression in rat mammary carcinoma cells after the administration of sumac in two doses. Data are expressed as mean ± SEM. Significant difference, * *p* < 0.05, ** *p* < 0.01, *** *p* < 0.001 vs. CONT, ^+^ *p* < 0.05 vs. SUM 0.1. Figure represents the expression of proteins quantified as the average percentage of antigen positive area in standard fields (0.5655 mm^2^) of tumor hotspot areas. The values of protein expression were compared between treated (SUM 0.1, SUM 1) and non-treated (control) carcinoma cells of female rats; >60 images for one marker were assessed.

Figure 4 summarizes evaluation of apoptosis (cytoplasmic cleaved caspase-3 expression, Bax and Bcl-2), proliferation (Ki67 expression), angiogenesis markers (vascular endothelial growth factor (VEGF) and vascular endothelial growth factor receptor 2 (VEGFR-2) expression, and anti-oxidant activity of *R. coriaria* (malondialdehyde (MDA) levels) in rat mammary carcinoma cells in vivo. Sumac at a higher dose increased caspase-3 expression by 80% (*p* < 0.05) and in a dose-dependent manner significantly increased Bax/Bcl-2 expression ratio by 98.5% (*p* < 0.01) and 125% (*p* < 0.001) versus controls. In addition, sumac dose-independently decreased Ki67 expression by 46% (*p* < 0.01) and 25% (*p* < 0.05), and dose-dependently decreased levels of MDA by 65% (*p* < 0.001) and 69% (*p* < 0.001) when compared to control specimens. Both parameters of angiogenesis were not significantly changed after chemoprevention with sumac. 

**Figure 5:**
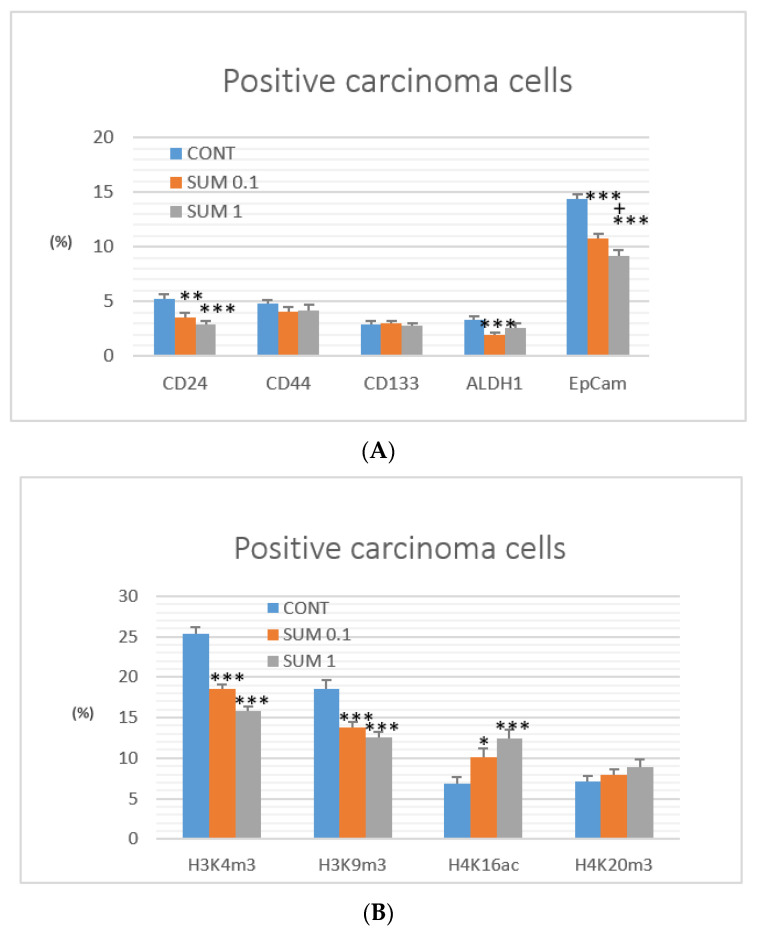
Immunoexpression of cancer stem cells (**A**) and epigenome (**B**) markers in rat mammary carcinoma cells after treatment with *R. coriaria*. Data are expressed as mean ± SEM. Significant difference: * *p* < 0.05, ** *p* < 0.01, *** *p* < 0.001 vs. CONT, ^+^ *p* < 0.05 vs. SUM 0.1. The values of protein expression were compared between treated (SUM 0.1, SUM 1) and non-treated (control) carcinoma cells of female rats; at least 60 images for one marker were analyzed.

Evaluation of CSCs parameters in rat carcinoma cells demonstrated a dose-dependent significant decrease in CD24 and EpCam expression in treated groups, when compared to the control group. CD24 was decreased by 31.5% (*p* < 0.01) and 43.5% (*p* < 0.001) and EpCam by 25.5% (*p* < 0.001) and 36% (*p* < 0.001). Expression of ALDH1 was decreased dose-independently by 42.5% (*p* < 0.001) and 22.5% (*p* = 0.11) in groups treated with sumac versus control. Expression of the remaining CSCs parameters (i.e., CD44 and CD133) did not significantly differ between experimental groups (Figure 5A).

Histone 3/4 post-translation chemical modifications in rat mammary carcinoma cells caused by sumac in lower dose showed decreases of H3K4m3 by 27% (*p* < 0.001) and H3K9m3 by 26% (*p* < 0.001) and increase of H4K16ac by 46% (*p* < 0.05) versus control group. Higher dose of sumac decreased the same parameters of H3 chemical modulations by 38% and 32% (both levels *p* < 0.001) and increased H4K16ac by 79% (*p* < 0.001) in comparison with control group. Alterations in H4K20m3 levels in treated groups were not significant when compared to the untreated group (Figure 5B).

**Figure 6:**
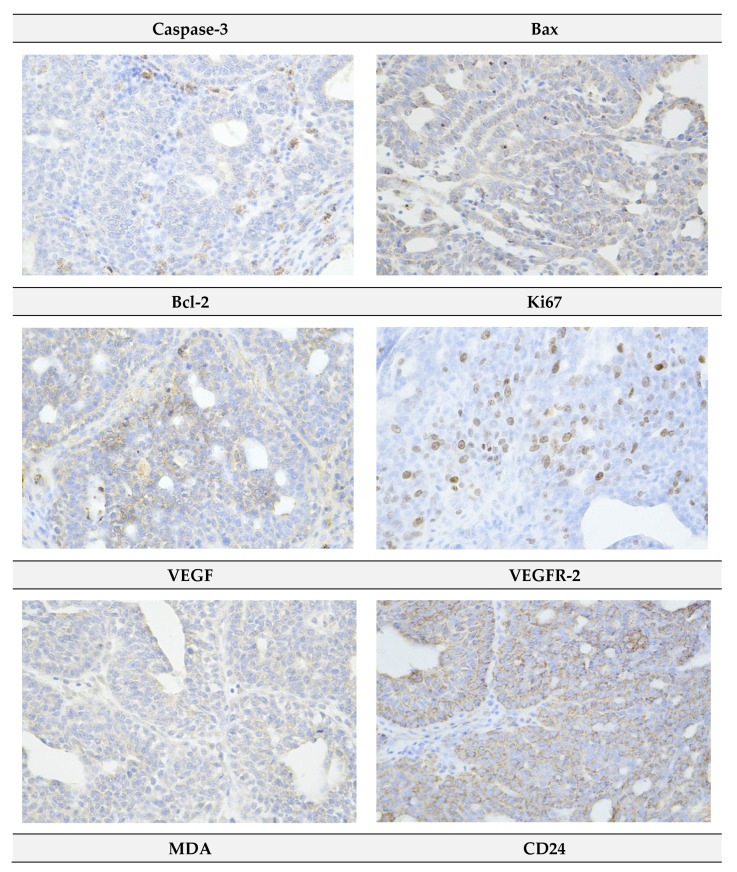

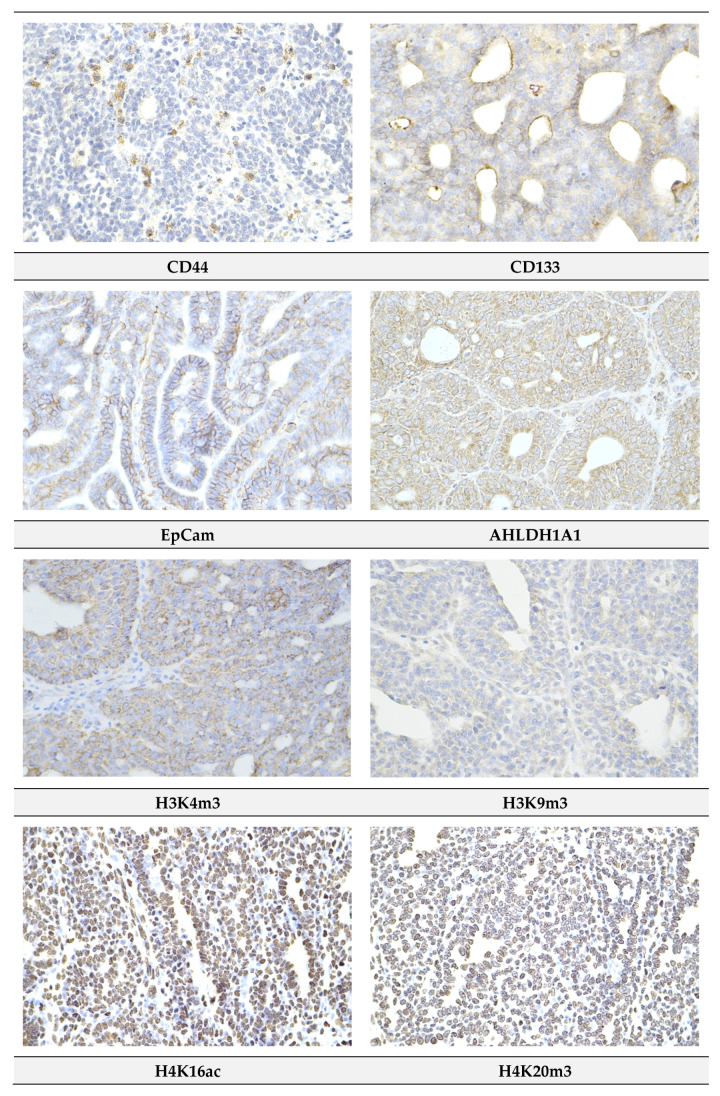

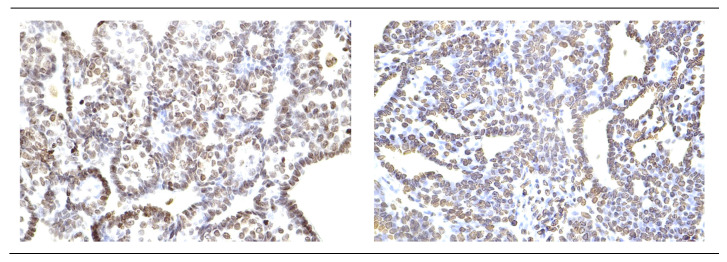
Representative images of expression of cleaved caspase-3, Bax, Bcl-2, Ki67, VEGFA, VEGFR-2, MDA, CD24, CD44, ALDH1A1, EpCam, H3K4m3, H3K9m3, H4K20m3, H4K16ac in rat carcinoma tissue of mammary gland. For detection, polyclonal caspase-3 antibody (Bioss, Woburn, MA, USA), polyclonal Bax and Bcl-2 antibodies (Santa Cruz Biotechnology, Paso Robles, CA, USA), monoclonal Ki67 antibody (Dako, Glostrup, Denmark), monoclonal VEGFA and VEGFR-2 antibodies (Santa Cruz Biotechnology, Paso Robles, CA, USA), polyclonal CD24 antibody (GeneTex, Irvine, CA, USA), polyclonal CD44 antibody (Boster, Pleasanton, CA, USA), polyclonal ALDH1A1 antibody (ThermoFisher, Rockford, IL, USA), polyclonal MDA, EpCAM, H3K4m, H3K9m3, and H4K20m3 antibodies (Abcam, Cambridge, MA, USA) and monoclonal H4K16ac antibody (Abcam, Cambridge, MA, USA) were applied; final magnification: ×400.

Representative pictures of the expressions of cleaved caspase-3, Bax, Bcl-2, Ki67, VEGFA, VEGFR-2, MDA, CD24, CD44, CD133, ALDH1A1, EpCam, H3K4m3, H3K9m3, H4K16ac, and H4K20m3 in rat mammary carcinomas are shown in Figure 6.

### 2.5. miRNA Expression

**Figure 7:**
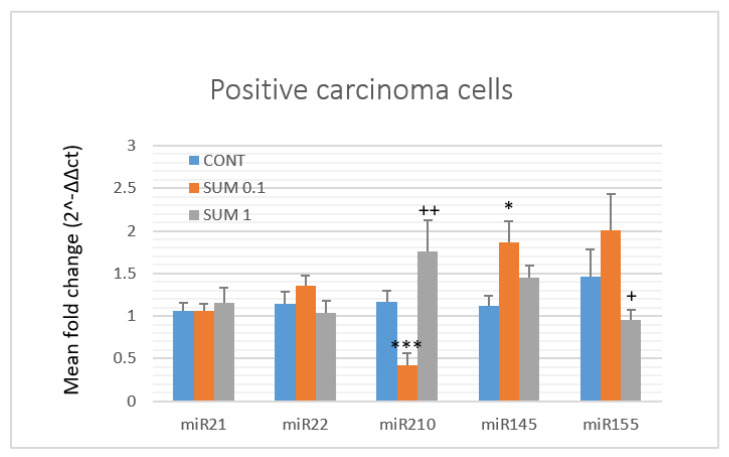
Relative miRNA expression of miR21, miR22, miR34a, miR210, miR145, and miR155 in rat mammary carcinomas. MiR-191-5p was selected as the internal control miRNA to normalize the cDNA levels of the samples. Data are expressed as mean ± SEM. Significant difference, * *p* < 0.05, *** *p* < 0.001 vs. CONT, ^+^ *p* < 0.05, ^++^ *p* < 0.01 vs. SUM 0.1.

With the aim to more precisely evaluate the mechanism of anticancer action of *R*. coriaria,** we analyzed the expression levels of five well-validated miRNAs (from both preclinical and clinical cancer research) in rat mammary cancer samples in vivo (Figure 7). In the lower dose treatment group, sumac significantly decreased expression of oncogenic miR210 by 64% (*p* < 0.001) and increased tumor-suppressive miR145 by 67% (*p* < 0.001) when compared to the controls. When comparing the SUM 0.1 vs. SUM 1 groups, we found a significant up-regulation of miR210 by 76% (*p* < 0.01), and down-regulation of oncogenic miR155 by 52.5% (*p* < 0.05). However, both doses of *R*. coriaria** did not significantly alter the expressions of oncogenic miR21 and miR155 and tumor-suppressive miR22 compared to the control group.

### 2.6. Gene Promoter Methylations Status

**Figure 8:**
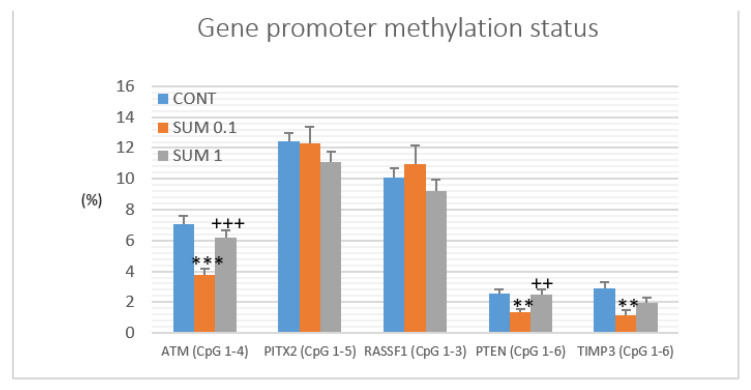
Promoter methylation status of ATM, PITX2, RASSF1A, PTEN, and TIMP3 genes in mammary carcinoma specimens in rats. Methylation status was appointed from all analyzed CpG isles of mentioned gene promoters (the number of analyzed isles is shown in the brackets). Significant differences, ** *p* < 0.01, *** *p* < 0.001 vs. CONT group and ^++^ *p* < 0.01, ^+++^ *p* < 0.001 vs. SUM 0.1 group.

The methylation status of five tumor-suppressor gene promoters was assessed: *ATM* including four evaluated CpG sites (CpG 1–4), *PITX2* (CpG 1–5), *RASSF1* (CpG 1–3), *PTEN* (CpG 1–6), and *TIMP3* (CpG 1–6) (Figure 8). We analyzed twenty rat tumor samples for each experimental group. *R. coriaria* administered at a lower dose significantly reduced total methylation status of ATM gene promoter by 46% (*p* < 0.001), PTEN gene promoter by 48.5% (*p* < 0.01), and TIMP3 gene promoter by 60% (*p* < 0.001) when compared to the control group. Analyzing all five parameters, sumac treatment at the higher dose did not show any significant changes on methylation status in comparison with the control group. Evaluating the sumac (0.1) group, our data showed significant decreases by 39% (*p* < 0.001) in the ATM gene promoter and 47.5% (*p* < 0.01) in the PTEN gene promoters when compared to the SUM 1 group (Figure 8).

### 2.7. Physiological In Vivo Effects

We did not find any significant differences in body weight gain in either rats or mice in the final week of both experiments. However, we have revealed a slight increase in the food intake of treated rats by 1.2 g (SUM 0.1, *p* < 0.05) and 0.9 g (SUM 1, *p* > 0.05) in comparison with the control group (16.4 g of diet/rat/day). In rats, continuous administration of sumac during 14 weeks was well tolerated. We did not observe any macroscopic organ changes evaluating liver steatosis, hepato/splenomegaly, or gastritis. Animals were found without hematopoietic disorders and other undesirable effects such as changes in vitality, hair, and mucosa. The mean dose of sumac per rat per day represented 17.26 mg in the SUM 0.1 group and 175.7 mg in the SUM 1.0 group. In mice, the daily mean doses of sumac were calculated as 7.0 mg (SUM 0.1) and 73 mg (SUM 1.0), respectively.

### 2.8. In Vitro Assessment on MCF-7 and MDA-MB-231 Cells

**Figure 9:**
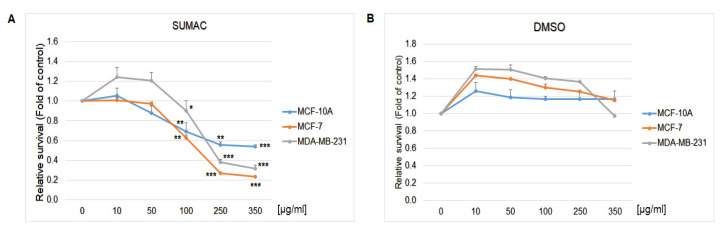
Relative survival of MCF-7, MDA-MB-231, and MCF-10A cells treated with (**A**) SUM (10–350 µg/mL) or (**B**) DMSO vehicle and analyzed by resazurin metabolic assay. Data were obtained from three independent experiments and significant differences were marked as * *p* < 0.05, ** *p* < 0.01, *** *p* < 0.001, vs. control cells (untreated).

The resazurin metabolic assays was used to evaluate the antiproliferative effect of sumac using MCF-7 and MDA-MB-231 breast cancer cell lines as well as MCF-10A, a non-cancer mammary gland epithelial cells. Results showed that sumac significantly decreased metabolic activity, followed by decreased cell survival in a dose-dependent manner in both tested cancer cell lines (Figure 9) with IC_50_ values 155 and 215 µg/mL, respectively. In cell lines comparison, MDA-MB-231 cells showed significantly less sensitivity to sumac treatment as MCF-7 cells. Furthermore, high doses of sumac treatment on healthy MCF-10A epithelial cells showed significantly higher viability and survival trend when compared with both cancer cell lines (IC_50_ = 350 µg/mL).

**Figure 10:**
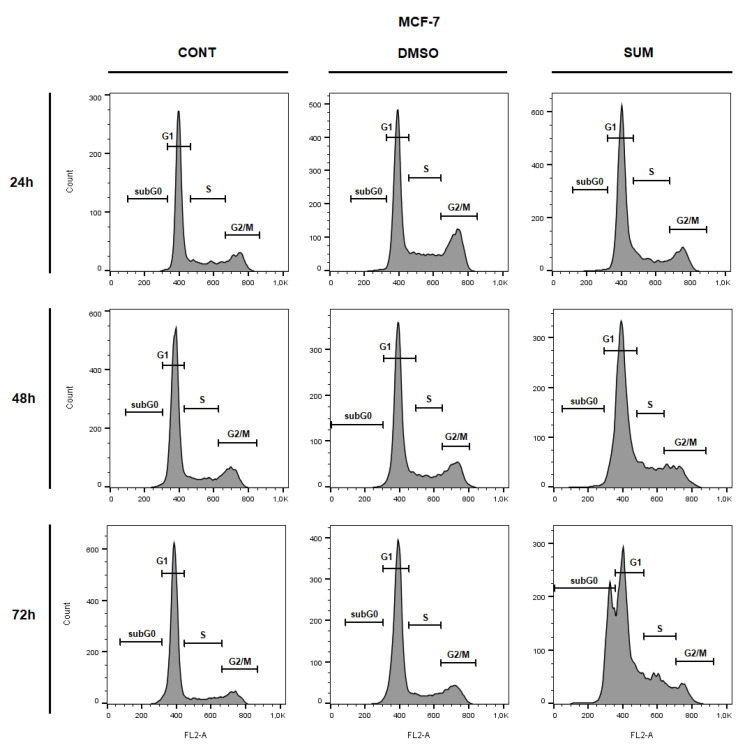
Representative diagrams of cell cycle distribution in MCF-7 cells after SUM treatment (155 µg/mL).

**Figure 11:**
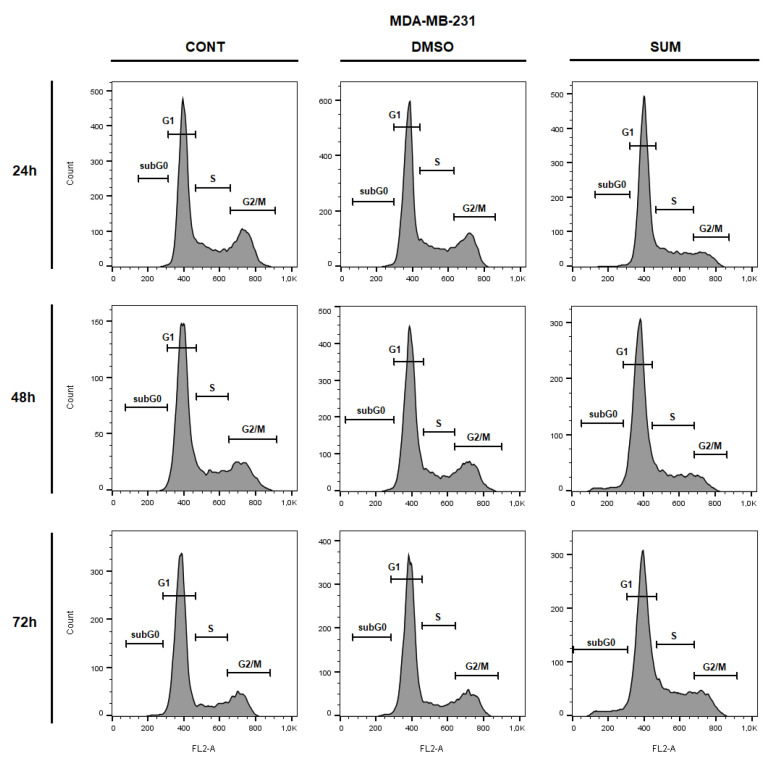
Representative diagrams of cell cycle distribution in MDA-MB-231 cells after SUM treatment (215 µg/mL).

The flow cytometric analyses of MCF-7 and MDA-MB-231 cells after sumac treatment were carried out after 24, 48, and 72 h. Evaluation of cell cycle progression (Table 3 and Table 4; Figure 10 and Figure 11) after sumac treatment in MCF-7 and MDA-MB-231 cells showed G1 cell cycle arrest after 24 h, which weakened in a time-dependent manner (after 48 h). Moreover, in MCF-7 cells we recognized apoptotic population with fractionated DNA (sub-G0/G1) after 72 h of sumac treatment and parallel decrease of cells in G1 phase of cell cycle. In MDA-MB-231cells, sumac treatment induced S phase cells accumulation after 72 h. In addition, we found delayed increase of cells in sub-G0/G1 population with proportional decrease in G1 phase slightly on 72 h. 

**Figure 12:**
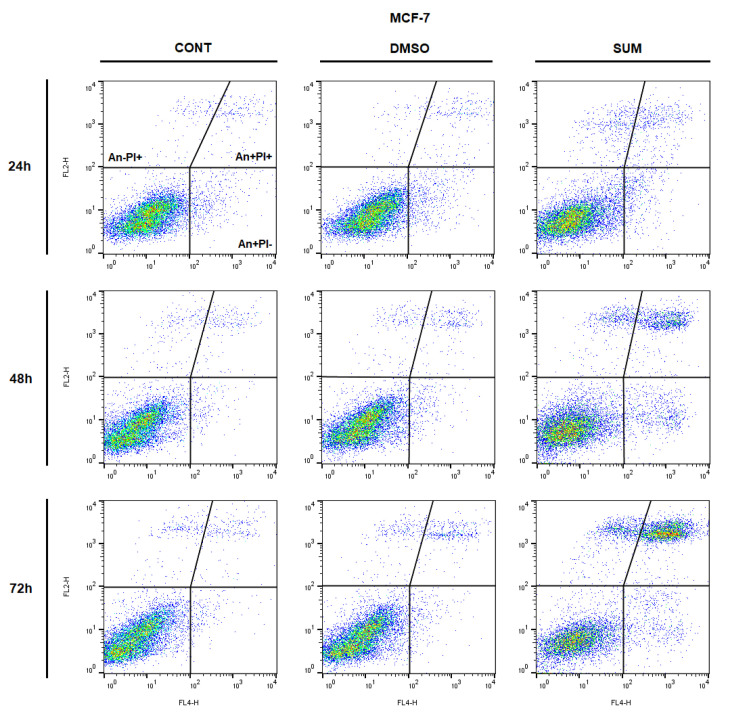
Representative diagrams of apoptotic cell diversification in MCF-7 cells after SUM treatment (155 µg/mL).

**Figure 13:**
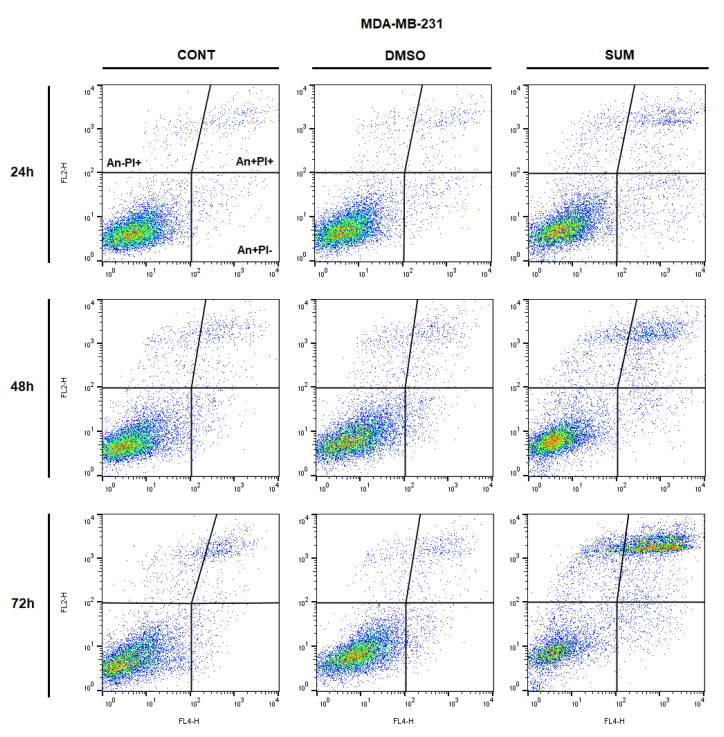
Representative diagrams of apoptotic cell diversification in MDA-MB-231 cells after SUM treatment (215 µg/mL).

The analysis of annexin V positivity, as a marker of programmed cell death induction, showed significant phosphatidyl serine (PS) externalization shortly after 24 h of sumac treatment in MDA-MB-231 cells and delayed (48, 72 h) increasing of PS in MCF-7 cells (Table 5 and Table 6; Figure 12 and Figure 13). Annexin V assay also showed diversification of cell population mostly in late apoptotic phase (An+PI+) with increasing An-cells (death/necrotic) at 72 h after sumac treatment. 

**Figure 14:**
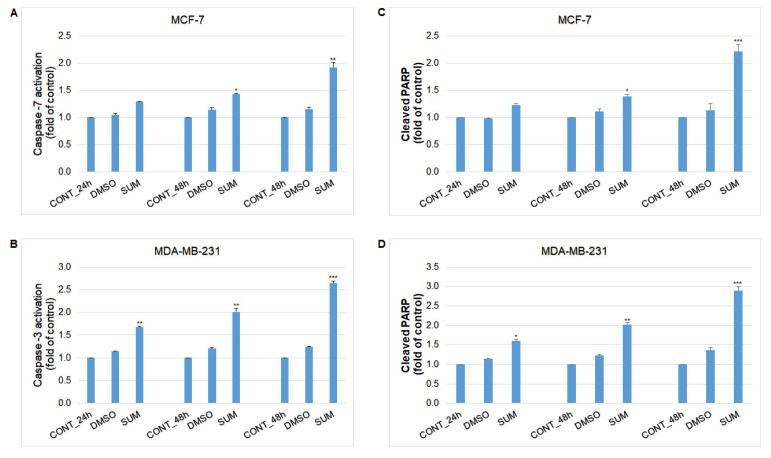
Effect of SUM treatment (155 or 215 µg/mL) on: (**A**) caspase-7 (MCF-7), (**B**) caspase-3 activation (MDA-MB-231), and PARP cleavage in both cell lines (**C**,**D**) analyzed by flow cytometry. Data were obtained from three independent experiments and significant differences were marked as * *p* < 0.05, ** *p* < 0.01, *** *p* < 0.001, versus control cells (untreated).

Caspase-dependent form of cell death in MCF-7 and MDA-MB-231 cells was confirmed after analysis of caspase-7 or caspase-3 activation (Figure 14). Both cell lines showed time-dependent trend of caspases activation after sumac treatment, leading to similar PARP cleavage rate (Figure 14). Analyses also showed delayed activation of caspases and PARP cleavage in MCF-7 cells (48–72 h) in comparison with MDA-MB-231 cells (shortly after 24 h).

**Figure 15:**
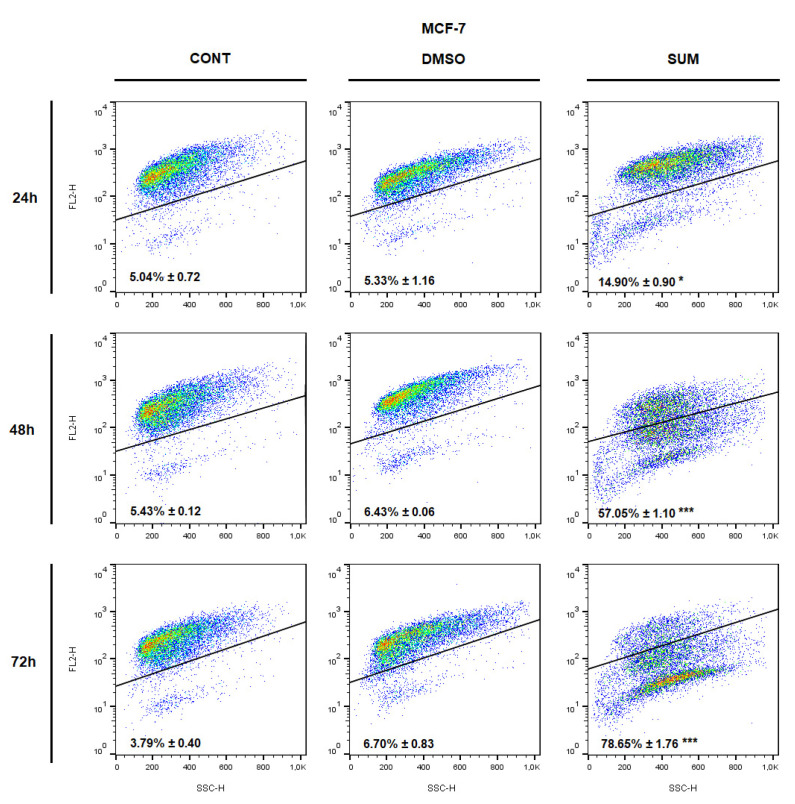
Mitochondrial membrane potential (MMP) changes in MCF-7 cells after SUM (155 µg/mL) treatment. Data were obtained from three independent experiments. Significant changes, control vs. SUM-treated cells * *p* < 0.05, *** *p* < 0.001.

**Figure 16:**
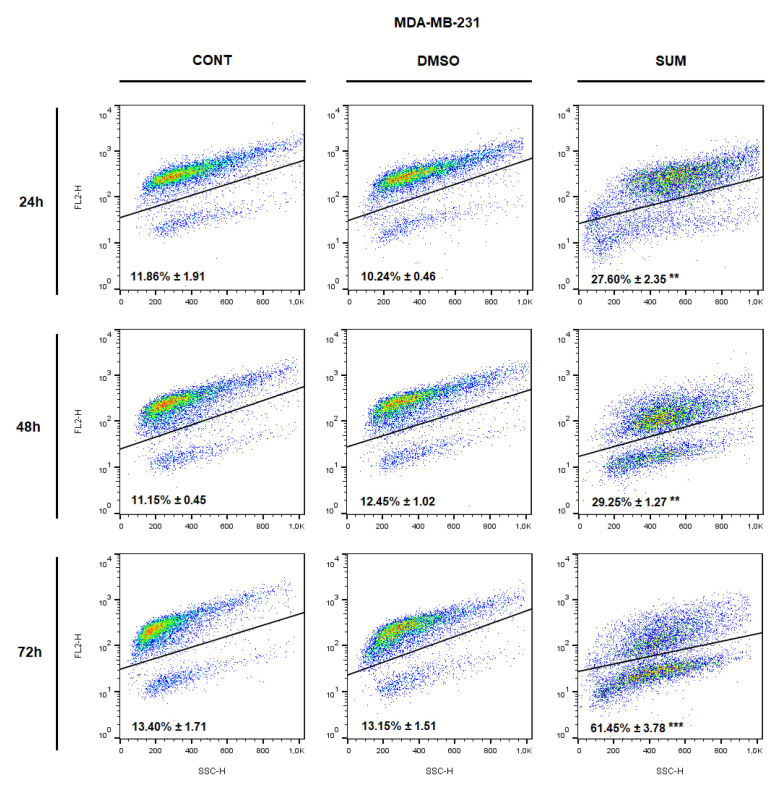
Mitochondrial membrane potential (MMP) changes in MDA-MB-231 cells after SUM (215 µg/mL) treatment. Data were obtained from three independent experiments. Significant changes, control vs. SUM-treated cells ** *p* < 0.01, *** *p* < 0.001.

**Figure 17:**
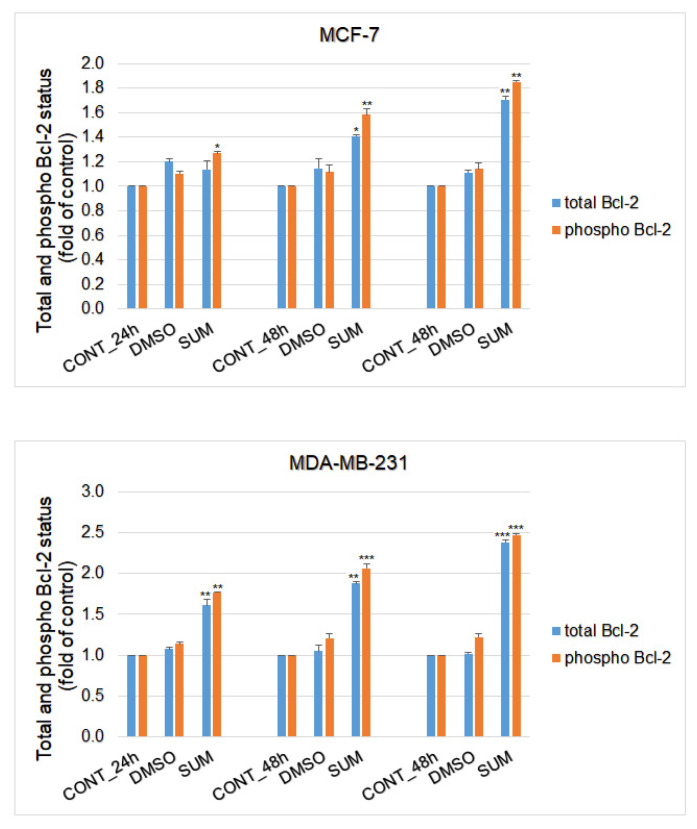
Flow cytometric analyses of anti-apoptotic mitochondria-associated protein Bcl-2 (total and phospho) expression after SUM treatment in MCF-7 (155 µg/mL) and MDA-MB-231 (215 µg/mL) cells. Results are expressed as mean values of three independent experiments. Significant changes, control vs. SUM-treated cells * *p* < 0.05, ** *p* < 0.01, *** *p* < 0.001.

Furthermore, time-dependent depletion of MMP in both MCF-7 and MDA-MB-231 cells occurred after sumac treatment (Figure 15 and Figure 16). Due to mitochondrial stress after sumac treatment, release of anti-apoptotic Bcl-2 (Figure 17) protein complexes from mitochondria to cytosol occurred significantly soon after 24 h in MDA-MB-231 cells and was delayed in MCF-7 cells (48 h). Analysis of phosphorylation status clearly showed deactivation in anti-apoptotic activity of Bcl-2 (Figure 17) and thus clearly demonstrates activation of mitochondrial apoptosis pathway in the same time. In comparison, MDA-MB-231 cell line showed significantly stronger and accelerated Bcl-2 release and deactivation after sumac treatment compared to MCF-7 cells. In MCF-7 cells, besides insignificant Bcl-2 release, Bcl-2 phosphorylation occurred shortly after 24 h of sumac treatment.

## 3. Discussion

Clinical trials evaluating plant-derived compounds against cancer are still in infancy, despite the fact of an overwhelming large number of these molecules currently under development [28]. Phytochemicals, their derivatives, or whole plant foods represent promising options in clinical practice to improve treatment efficiency in cancer patients, suppress adverse reactions induced by conventional therapies, and decrease cancer risk in high-risk individuals [29,30,31]. With the aim to reveal new perspective medicinal plants with significant oncostatic activities, we focused to assess the therapeutic and preventive potential of *R. coriaria* in breast cancer animal models and human cell lines.

The efficient dose of anti-cancer therapeutics is specific for different mammalian species. The doses of sumac applied in this experiment were derived from expertise with the allograft and chemically-induced breast carcinoma rodent models [21,22,23,24,25,26]. In the murine allograft study, a higher dose of sumac significantly reduced the volume of 4T1 tumors by 27%. This result was accompanied with a dose-dependent decrease in mitotic activity index by 36.5% and 51% in treated groups when compared to control cancer specimens. In our recent studies, using the same 4T1 mice model, *C. zeylanicum* (1% *w*/*w*) decreased volume of 4T1 tumors by 44 % and *T. vulgaris* (1% *w*/*w*) by more than 80%, when compared to control groups [21,22]. Previous works from our laboratory have described significant therapeutic effects of sumac, cinnamon, and thyme, which are comparable with the therapeutic activities of synthetic drugs evaluated in the 4T1 breast carcinoma model in mice [32,33,34]. Chemoprevention of NMU-induced rat mammary carcinogenesis by sumac in this study demonstrated significant reduction of tumor incidence by 20% (SUM 1% *w*/*w*) and non-significant reduction of tumor frequency by 29% (SUM 0.1% *w*/*w*) compared to controls. In addition, sumac significantly improved histopathological characteristics of rat mammary carcinomas (high-grade/low-grade ratio) in both treated groups in dose dependent manner by 66 and 73% vs. the control group. However, contradictory data showed a slight non-significant increase in tumor volume in treated groups versus control in the 4T1 model. This observation points to the fact that treatment potential of sumac is dependent on cancer type. Robust chemopreventive effects of plant foods (dark fruits, herbs, or spices) in rat mammary carcinogenesis were found in our laboratory [21,22,23,24,25,26,27] and by other authors [35,36,37,38]. The dominant substances present in sumac are phenolics, particularly hydrolysable tannins derived from gallic acid, that have previously been described as molecules with significant tumor suppressive activities against various cancer cell lines, including breast cancer [39]. The direct bioavailability of hydrolysable tannins are generally found to be limited; however, it was shown that they are extensively metabolized in the gut and their effect can be mediated by their metabolites [40]. From this information, we believe that dominant metabolites from methanolic extract of *R. coriaria* were present in plasma of used rodents after consumption of dietary administered sumac in this experiment. Numerous papers describe significantly higher anticancer efficacy of natural phytochemical complexes present in whole foods, in comparison to isolated plant-derived molecules, and has been concluded in our recent comprehensive and critical review paper [41]. Targeting multiple signaling pathways linked with carcinogenesis by dozens of bioactive molecules present in plant “superfoods” may represent perspective and logical biomedical approaches in clinical oncology practice. However, the data translation from preclinical oncology research to clinical use is not easily applicable. In this regard, several issues must be resolved before introducing plant foods into clinical oncology: (1) phytochemicals or their active metabolites must achieve sufficient levels in human plasma using progressive methods such as nanotechnology; (2) data relating to pharmacokinetic parameters (e.g., absorption, excretion) of numerous phytochemicals are needed; (3) identification of safe and optimal doses of specific plant natural substances; (4) data concerning drug combinations with the aim to decrease the conventional medications dosing are required; (5) determination of the impact on various cell signaling pathways; (6) data about the re-sensitizing of the chemotherapy/radiotherapy-resistant cancers by plant foods; (7) effect on reduction of cancer cell invasion/metastasis and risk of disease relapse; and (8) finally, understanding the mechanisms linked with the individual characteristics of patients with the aim to develop a more personalized medical approach using plant foods.

We believe that the tumor suppressive activities of sumac found in this study are based on the proapoptotic, antiproliferative, antiangiogenic, or antioxidant mode of action induced by specific mixture of plant secondary metabolites. Apoptosis, as a programmed cell death, can be initiated through one of two pathways—Ωintrinsic or extrinsic. In this study, we evaluated intrinsic mechanisms using in vivo and in vitro models. This pathway is modulated by two groups of molecules, Bcl-2 and Bax. Increased Bax/Bcl-2 ratio up-regulates caspase-3 activity and subsequently triggers programmed cell death in cancer cells [42]. Numerous literature have documented an important regulatory role of phytochemicals in Bax/Bcl-2/caspase-3 signaling in breast carcinogenesis [43,44,45]. This experiment clearly shows a significant dose-dependent increase in the Bax/Bcl-2 ratio in rat mammary tumors induced by sumac treatment, which correlates with an increase in cleaved caspase-3 expression in the treated group and higher sumac dose. Similarly, in our recent chemopreventive studies using rat models, we described significant correlation between increase in Bax/Bcl-2 ratio and an increase in caspase-3 expression in mammary carcinoma cells induced by dark fruit peel, oregano, clove buds, and cinnamon [21,23,24,25]. The apoptotic effects of sumac in this study were also documented in our parallel in vitro analyses using MCF-7 and MDA-MB-231 BC cell lines. Sumac treatment induced different dose-dependent antiproliferative activity in both cell lines. On the contrary, the comparative study with non-cancerous breast cell line MCF-10A showed significantly lower effect after sumac exposure. The IC 50 calculation revealed more than 1.5x or 2x higher values compared to MCF-7 or MDA-MB-231 cells. We observed that the susceptibility of the breast cancer and non-cancerous cells towards growth inhibition induced by sumac extract was differentially affected. It is known that MCF7 and MDA-MB-231 cells are both invasive ductal/breast carcinoma cells with many phenotypic/genotypic differences. The MCF7 cells express the epithelial phenotype in contrast to MDA-MB-231 that are more mesenchymal [46] and have also been documented for their multidrug resistance [47]. Minimal one of the mentioned cell-based difference may contributed to different dose-dependent action of sumac extract we recorded. The results from this study demonstrated the ability of sumac treatment to decrease MCF-7 cell viability. These changes were associated with a G1 cell cycle arrest and an increase in the population of MCF-7 cells in the sub-G0/G1 DNA content. In MDA-MB-231 cells, we revealed a G1 cell cycle arrest but delayed increase of cells in the G0/G1 phase and increase in S phase of the cell cycle. These data indicate the block of cell cycle progression and shortly delayed induction of apoptosis in MCF-7 and MBA-MD-231 cells after the treatment with sumac extract. In addition, we observed apoptosis or necrosis in the MDA-MB-231 and MCF-7 cells using annexin V/PI staining. Furthermore, we observed a decrease in the mitochondrial membrane potential and inactivation of Bcl-2 protein induced by sumac extract. It is generally accepted that there is a close relationship between matrix metalloproteinases (MMP) and activity of proteins of Bcl-2 family [48]. Decrease in Bcl-2 expression (or its inactivation via phosphorylation) leads to the loss of MMP, cytochrome c release, and caspases activation with subsequent apoptosis induction [49,50]. Results of our experiments also showed activation of caspase-7 or caspase-3 and PARP cleavage (other characteristic signs of apoptosis) in sumac-treated cells. Results derived from this experiment show that sumac represents a potentially effective apoptosis-inducing plant food in breast cancer models.

A plethora of papers from preclinical research have documented the antiproliferative activities of phytochemicals in breast carcinoma models [51,52,53]. Several pathways/mechanisms such as COX-2, Nrf2, poly-ADP-ribosylation, Hedgehog, Wnt, Plk1, STAT3, NF-κB, PI3 kinase, or epigenetic modulations are implied in the modulation of cancer proliferation [54]. Ki67 protein is known as an important proliferation biomarker. The immunohistochemical evaluation of the expression levels of this marker in rat mammary carcinoma cells in vivo showed a significant down-regulation in both groups treated with sumac vs controls. In addition, histopathological assessment of mice 4T1 tumors demonstrated a significant decrease in the mitotic activity index that was found in both groups treated with sumac. Using resazurin viability assays, we confirmed our data obtained from in vivo rodent studies. The sumac methanol extract reduced the viability and metabolic activity of MCF-7 and MDA-MB-231 cell lines. The anti-proliferative effect of sumac extract in human breast adenocarcinoma cells was also validated by the blocking cell cycle progression in G1 or S phase. However, previous analyses from our laboratory using *Ch. pyrenoidosa* and *T. vulgaris* haulm documented discrepancies using different breast cancer models [22,27]. Heterogeneous genotypes and phenotypes displayed by malignancies in animal and cell culture are responsible for different therapeutical activity (i.e., impacting on cell proliferation) of phytochemicals present in plant-derived foods.

Analyses focused on the therapeutic possibilities of new drugs affecting cell signaling of neovascularization (e.g., VEGF, FGF, EGF, HGF, neuropilins, integrins, tyrosine kinases receptors) represent an important area in oncology research [55]. Natural plant foods rich in bioactive molecules that significantly affect the activity of VEGF/VEGFR complex and subsequent cell neoangiogenesis signalizations have been shown to suppress carcinogenesis [56,57,58]. However, we did not observe any significant alterations in the expression levels of VEGF and VEGFR-2 induced by sumac in our in vivo studies. Conversely, in our two last studies using in vivo rat models, we found a decrease in VEGFR-2 expression after administration of thyme [22], and a reduction in VEGF expression in cancer cells after cinnamon treatment [21]. Similarly, our previous studies involving in vivo rat models demonstrated that natural mixtures of plant bioactive molecules present in plant foods have significant anti-angiogenic activities in rat mammary cancer tissue [23,24,26]. However, the determination of the exact role of natural plant foods neoangiogenesis during carcinogenesis warrant further and more complex evaluations, specifically related to the individual mechanisms and cell signalization of the process of angiogenesis and vasculogenesis.

Intracellular oxidative stress is a consequence of imbalanced cellular redox status, resulting in oxidative damage to key cellular biomolecules such as DNA, proteins, and lipids. Such alterations in the cell are principally included in the etiopathogenesis of cancer [59,60]. Antioxidant mechanism of action exerted by naturally occurring substances may be important in the prevention of DNA damage, and thus their use is appropriate in the chemopreventive model of NMU-induced mammary carcinogenesis in rats. Perennial outcomes from our laboratory revealed that natural plant foods, such as cinnamon, young barley, fruit peel polyphenols, clove buds, and thyme, significantly reduced oxidative damage of cellular lipids or proteins [21,22,23,25,26]. Cellular levels of MDA, a well-established and frequently used biomarker of oxidative damage of lipids by free radicals [61], has been analyzed by our team in previous experiments. Results from this study showed a significant decrease in MDA levels in rat cancer cells, alluding to the antioxidant activity of *R. coriaria* and its genoprotective effects in the cell. Pro-oxidative effects of a broad spectrum of phytochemicals/extracts in cancer cells, have been associated with the application of very high doses of drugs using in vitro experimental conditions [62] that do not consistently mimic the physiologically relevant state of in vivo systems.

Oncology research showed that phytochemicals targeting signal pathways, such as Wnt, Notch, or Hedgehog, adversely affect the activity of cancer stem cells (CSC) that are involved in the processes of cancer apoptosis and drug resistance [63,64]. Several established markers of breast cancer CSCs, including CD44^+^/CD24^−/low^ phenotype and CD133, ALDH1, EpCAM, and nestin overexpression, have been correlated with poorer prognosis diagnosed patients [65,66]. In addition, CD24, CD44, CD133, EpCAM, and ALDH1 have been documented as beneficial CSC markers in the chemically-induced rat mammary carcinoma model [67,68]. The comparative preclinical studies found that plant foods with the natural mixtures of bioactives show higher anti-cancer potential (including the anti-CSCeffects) compared to isolated plant-derived molecules [41]. In this experiment, sumac significantly reduced expression levels of CD24, ALDH1, and EpCam in cancer cells. Using the same animal model, we found significant anti-CSC effects of cinnamon, thyme haulm, clove buds, and oregano haulm [21,22,23,24]. Moreover, cancer research has shown significant anti-CSC activities of plant-derived molecules against a wide variety of cancer types [69]. It is assumed that these activities are mediated through modulation of multiple cell mechanisms/signaling processes that demand an urgent need for in-depth evaluation within oncology research [70].

The impact of phytochemicals on the cancer epigenome is a highly relevant topic in oncology research [71,72]. Epigenetic modifications include global methylation status (e.g., in promoter regions of oncogenes and tumor-suppressor genes), histone chemical modifications, and non-coding RNA-associated multi-gene control [3,73]. It is well documented that phytochemicals and/or plant foods affect the activity of DNA methyltransferases and other epigenetic mechanisms such as posttranslational modifications of histone molecules [74]. Epigenetic biomarkers evaluated in this study represent validated and frequently used diagnostic and prognostic parameters in clinical oncology. Down-regulation of these markers is associated with breast cancer disease [75] or experimental mammary carcinogenesis [22]. The analysis of the TSG promoters‘ methylation level of standardized CpG islands in cancer tissue revealed significant decreases of *ATM*, *PTEN*, and *TIMP3* genes after sumac treatment. In this study, significant decreases in gene promoter methylation statuses in cancer cells were observed only after treatment with lower sumac dose, thus supporting the potential chemoprevention outcome of this study, by which a lower dose of sumac showed a more pronounced efficacy compared to a higher sumac dose (assessing the most sensitive parameter—tumor frequency). Higher efficacy of the lower sumac dose in the chemoprevention study could be explained by excessive dose, leading to increased oxidative stress in normal cells and consequently to general toxicity in the animals. Our previous experiments using the same rat model revealed that the natural bioactives present in *C. zeylanicum*, *T. vulgaris*, and *S. aromaticum* induced significant down-regulations in the methylation patterns of specific gene promoters (cinnamon: ATM and TIMP3; thyme: *ATM, RASSF1, PTEN,* and *TIMP3;* and clove: *RASSF1* [21,22,23]). Demethylation activities of plant foods on TSG promoters regions have also been documented other research groups. Dietary administration of black raspberries resulted in demethylation of *WIF1*, *SOX17*, and *GKI* in in vivo colon cancer models [76]. In the rat esophageal squamous cell papilloma model, black raspberries administration was effective during demethylation of the *Sfr4* gene promoter region [77].

Analyses of alterations in posttranslational histone modifications that are associated with various types of chronic diseases (including cancer) offers the progressive medicinal approach that could be applied as predictive and prognostic markers [78]. Targeting the histone-modifying enzymes by plant-derived bioactives represents an important challenge for preclinical and clinical research. In this study, sumac significantly decreased by dose-dependent manner H3K4m3 and H3K9m3 expression levels and dose-dependently increased H4K16ac levels in rat cancer specimens. In the same rat model, we found positive changes in histone modifications after cinnamon [21], clove buds [23], and thyme [22] treatments. Other authors described a reduction in H4R3me2s and H3K27me3, and an increase in H3K9ac and H3K27ac levels induced by resveratrol in MCF-7 and MDA-MB-231 cells [79]. This outcome was correlated with cytotoxic activities and increased expression of TSG such as BRCA1, p53, and p21 induced by resveratrol [79]. In another in vitro study using MCF-7 and MDA-MD-231 cells, decreased levels of histone deacetylase induced by the combination treatment with sulforaphane and withaferin A were associated with cytotoxic and proapoptotic effects and suppression of cell cycle progression due to linked up-regulations in histone methylations [80]. Despite the fact that the precise mechanisms by which phytochemicals affect DNA methylation status and posttranslational histone modifications are not fully understood, numerous data from oncology research demonstrate that isolated phytochemicals and/or plant foods represent a perspective tool to manage cancer [74,81]. Better understanding of the global patterns in the gene promoter methylation status and posttranslational histone modifications with the specific consequences for the structure and activity of nuclear chromatin may uncover new molecular targets for dietary phytochemicals potentially applied within progressive anticancer therapies.

Regulation of functions and other genes during protein synthesis is regulated by miRNA. A number of phytochemicals have been described as molecules that efficiently affect miRNAs activity in the processes of carcinogenesis [82,83,84]. Numerous miRNAs have been applied as valid biomarkers in breast cancer diagnosis and prognosis. MiR21, miR155, miR210 represent oncogenic miRNAs. Whereas, miR22 and miR145 are described as positive regulators of tumor-suppressors in breast cancer research [85,86,87,88]. Data from this study revealed significant activity of the lower dose *of* sumac in rat cancer cells in vivo, as shown by decreased expression of oncogenic miR210 and increased tumor-suppressive miR145 when compared to controls. We did not find any changes in miRNA expression between the higher sumac dose and control group. Effectivity of lower sumac dose compared to the ineffectivity at a higher dose in this study correlates with the results observed in our chemoprevention study. In addition, significant effects of plant foods on miRNA expressions, using the same model, were recorded in our previous experiments [21,22]. Despite the previously mentioned positive results regarding the effect of plant foods on miRNA expression, little is known regarding the mechanism by which the phytochemicals affect this expression [73]. Deeper understanding of these molecular mechanism, using more extensive research, may be very useful for controlling multiple processes of carcinogenesis and more effective application of plant-derived bioactives in cancer therapy [89].

## 4. Materials and Methods

The experiments were approved by the Ethical Commission of the Jessenius Faculty of Medicine of Comenius University (Protocol No. EK1860/2016) and by the State Veterinary and Food Administration of the Slovak Republic (accreditation No. Ro-3239/15-221 and Ro-1640/17-221).

### 4.1. Animal Models

Female Sprague-Dawley rats at 5 weeks of age and weighing between 125–140 g (Charles River Laboratories, Sulzfeld, Germany) and female BALB/c mice (Velaz, Prague, Czech Republic) at 10 weeks of age and weighing between 17–19 g were used in the study. Rodents were acclimatized to controlled vivarium conditions with artificial light regimen (L/D 12: 12 h), temperature (23 ± 2 °C), and relative humidity (40–60%). Animals were fed a Ssniff^®^ diet (R-Z/M-Z low-phytoestrogen) (Soest, Germany), and drinking water was supplied ad libitum. Mammary gland carcinogenesis in rats was induced by *N*-nitroso-*N*-methylurea (NMU, Sigma, Deisenhofen, Germany). The carcinogen was administered by intraperitoneally injection (single dose of 50 mg/kg) on day 42, postnatal. This experimental approach mimics premenopausal women with increased risk for breast cancer pathogenesis. A syngeneic mouse model was used as a therapeutic model of breast cancer. 4T1 (mouse mammary adenocarcinoma) cells in the amount of 1 × 10^5^ cells/animal were inoculated by subcutaneous application into the abdominal mammary gland area.

The administration of sumac powder, obtained from *R. coriaria* fruits (SONNENTOR Kräuterhandels GMBH, Sprögnitz, Austria; country of origin—Iran), was used as a chemopreventive substance in rats one week prior to carcinogen administration, and lasted for 14 weeks consecutive weeks. In the allograft study, *R. coriaria* administration started on the day of 4T1 cells’ inoculation and lasted for 15 days. In both rat and mouse studies, *R. coriaria* was administered via diet (peels were processed by “cold pelleting procedure”) using two concentrations: (a) a low dose of 1 g/kg–0.1% (*w*/*w*), and (b) a high dose of 10 g/kg–1% (*w*/*w*). Rats/mice (totally 75/78 of animals, *n* = 25/26 animals per group) were randomly divided into three experimental groups: (1) control group without chemoprevention or therapy; (2) chemoprevention/therapy with *R. coriaria* at a lower dose (sumac 0.1%); and (3) chemoprevention/therapy with *R. coriaria* at a higher dose (sumac 1%). From the fifth week after NMU application, the rats were palpated weekly with the aim to check the presence, size, and location of each mammary tumor (palpable if tumor diameter is >0.4–0.5 cm). In mice, the tumor growth (volume) was monitored 3 times per week from the fourth day after inoculation of 4T1 cells. Dietary intake was measured 4 times in rats and 2 times in mice within 24 h periods during the study. Consequently, the mean daily doses of *R. coriaria* per rat and mouse in individual groups were measured. At the end of both studies, animals were euthanized by decapitation and mammary tumors were excised and measured.

### 4.2. Histopathological and Immunohistochemical Evaluations of Rat and Mouse Tumors

Formalin-fixation and paraffin-embedding of tissue samples of each mouse and rat adenocarcinomas were routinely performed. Rat mammary tumors were classified in accordance with the criteria for the standardized classification of mammary tumors. According to the additional parameter (grade of invasive carcinomas), rat mammary tumors were also divided into low-grade and high-grade carcinomas. The selection of categorization criteria (solidization, cell atypia, mitotic activity index, necrosis) was based on the standard diagnostic method of classification: the solidization was considered if >30% of tumor sample displays solid growth, high mitotic activity index of ≥10 mitosis is observed in 10 high power fields and necrosis if the occurrence of comedo (not infarct) was determined [90]. Tumors with ≥2 positive criteria were considered as high-grade carcinomas and tumors with ≤1 positive criterion were considered as low grade carcinomas. Mitotic activity index and all tumor area/necrosis ratio were assessed in mice tumors. The evaluation of metabolic parameters, which included total cholesterol, low-density lipoprotein cholesterol, very low-density lipoprotein cholesterol, high-density lipoprotein cholesterol, triacylglycerols, and glucose, in rat serum was performed using an Olympus AU640 (Olympus Optical, Tokyo, Japan) automatic biochemical analyzer.

The most relevant part of rat mammary tumor in paraffin block (that included the typing characteristics) with largest representation of vital tumor epithelial component (i.e., without regressive changes such as extensive necrosis) was chosen for immunohistochemical analysis. Indirect immunohistochemical method was used for the detection of markers selected for the mechanistic study performed on whole paraffin sections with the utilization of commercially available rat-specific antibodies (Abcam, Cambridge, MA, USA; Bioss, Woburn, MA, USA; Boster Biological Technology, Pleasanton, CA, USA; Dako, Glostrup, Denmark; GeneTex, Irvine, CA, USA; Santa Cruz Biotechnology, Paso Robles, CA, USA; Thermo Fisher Scientific, Rockford, IL, USA). Immunohistochemical staining (Autostainer Link 48 /Hermes/) was performed according to the manufacturer’s recommendations. The concentration used for each primary antibody was as followed: cleaved caspase-3 1:500 (catalog no. ab2302); Bax 1:200 (sc-526); Bcl-2 1:200 (sc-492); Ki-67 1:50 (M7248 01); VEGFA 1:150 (sc-57496); VEGFR-2 1:80 (sc-6251); MDA 1:1000 (ab6463); CD24 1:200 (gtx37755); CD44 1:200 (pa1021-2); CD133 1:150 (ab19898); ALDH1A1 1:500 (pa532127); EpCam 1:160 (ab71916); H3K4m3 1:500 (ab8580); H3K9m3 1:400 (ab8898); H4K20m3 1:300 (ab9053); and H4K16ac 1:200 (ab109463). A secondary staining system (EnVision, Dual Link System-HRP, cat. No. K060911, Dako North America, Carpinteria, CA, USA) was used to visualize primary antibodies using diaminobenzidine tetrahydrochloride as a substrate. Negative controls had the primary antibody omitted. A precise morphometric method was used to evaluate the immunohistochemically detected antigen expression. Sections were screened and Olympus BX41N was used for the microscopic analysis of digital images at magnifications of ×400. The protein expression was quantified as the average percentage of antigen positive area in standard fields (0.5655 mm^2^) of tumor cells hot spot areas. Three hot spots per tumor sample were analyzed using the morphometric method. QuickPHOTO MICRO software, version 3.0 (Promicra, Prague, Czech Republic), was used for the morphometric analysis of the digital images. The values were compared between treated (CIN 0.1 and CIN 1) and non-treated (control) tumor tissue specimens of female rats. At least 60 tumor samples for one marker (900 tumor slides for 15 markers) were analyzed.

### 4.3. Analysis of miRNA Expression

A commercial miRVana microRNA isolation kit (Thermo Fisher Scientific, Waltham, MA, USA) with detailed description contained in supplementary protocol was used for isolation of total RNA from tumor tissues. After that, RNA quantification was performed on NanoDrop ND-2000 spectrophotometer (Thermo Scientific, Wilmington, Delaware, USA) with subsequent reverse transcription achieved by TaqMan advanced miRNA cDNA synthesis Kit (Applied Biosystems, Life Technologies, Carlsbad, CA, USA). The samples of cDNA were stored at −20 °C for future application. The miRNA-specific TaqMan^TM^ advanced miRNA assays kit (Applied Biosystems, Life Technologies, Carlsbad, CA, USA) for tumor-suppressor miR-22, miR-34a, miR-210 and for the oncogenic target miR-21 was used for quantitative real-time PCR. According to relevant publications and protocol recommendations, as an internal control to normalize the levels of cDNA of samples was chosen miR-191-5p. AB7500 real-time system (Applied Biosystems, Life Technologies, Carlsbad, CA, USA) was used for quantitative real-time PCR reaction and all qPCR reactions were made in duplicate with consequent average of obtained Cq values.

### 4.4. DNA Isolation and Bisulfite Conversion

Fresh frozen tissue samples were mechanically disrupted using TissueLyser LT (Qiagen, Germany). Tissue samples (an average of 100 mg) were placed in the 2 mL precooled tube with 5 mm stainless steel beads (Qiagen, Germany). Biological material was homogenized in 200 μL of lysis buffer (Qiagen, Germany) for 1 min with an oscillation of 50 Hz. Subsequently, disrupted samples with the addition of 20 μL of proteinase K were incubated at 56 °C. Genomic DNA was isolated using DNeasy blood and tissue kit (Qiagen, Germany) as per the manufacture´s protocol. The concentration of DNA was determined by the Qubit™ 3.0 fluorometer (Thermo Fisher Scientific) with Qubit dsDNA BR assay kit (Thermo Fisher Scientific). Isolated DNA (in concentration at least 50 ng/μL) was modified by bisulfite conversion using EpiTect bisulfite kit (Qiagen, Germany) according to the supplementary protocol.

### 4.5. Determination of Methylated CpG Islands in the Promoter Regions (Pyrosequencing)

Pyrosequencing was used to determine the percentage of the methylation status of CpG islands localized in promotor regions of tumor suppressor genes TIMP3 (three CpG islands), PTEN (six CpG islands), RASSF1A (three CpG islands), PITX2 (five CpG islands), and ATM (four CpG islands) using predesigned methylation PyroMark CpG assays (Qiagen, Germany). Primer sequences are showed in the supplementary material of the publication. Briefly, 20 ng of bisulfite-treated DNA was used as a template in PCR reaction in the total volume of 25 μL (PyroMark PCR kit, Qiagen, Germany). Thermal cycling conditions were as follows: initial denaturation at 95 °C for 15 min followed by 45 cycles of amplification consisting of denaturation (94 °C, 30 s.), annealing (56 °C, 30 s.); extension (72 °C, 30 s.) with a final extension at 72 °C for 10 min. The PCR product was separated by gel electrophoresis and visualized on 1.75 % agarose gel. The PCR product was further analyzed according to the manufacture’s instructions available in a supplementary protocol using PyroMark Q96 ID System (Qiagen, Germany) with PyroMark Gold Q96 Reagents. Obtained data were interpreted by PyroMark Q96 software version 2.5.8 (Qiagen, Germany).

### 4.6. Cell Lines, Cell Cultures, and Experimental Design

In the in vitro experiments, human breast cancer cell lines MCF-7 (ER+, PR+, HER2−), MDA-MB-231 (ER− PR−, HER2−) and non-cancer MCF-10A (human mammary gland epithelial cells) were used. Breast cancer cells were cultured in DMEM medium (GE Healthcare, Piscataway, NJ, USA) or RPM1 1640 medium (Biosera, Kansas City, MO, USA) and non-cancer cells were cultured in DMEM F12 medium (Biosera, Kansas City, MO, USA) + supplemented with insulin, EGF- epithelial growth factor, HC-hydrocortisone (all Sigma, Steinheim, Germany). The growth medium was supplemented with 10% FBS (Gibco), antibiotic/antimycotic solution (1× HyClone™; GE Healthcare, Chicago, IL, USA) and cells were cultivated in an atmosphere containing 5% CO_2_ in humidified air at 37 °C. Before each experiments, the cell viability was estimated by trypan blue exclusion (≥95%).

For the flow cytometry experiments, the MCF-7 (3 × 10^5^) and MDA-MB-231 (1 × 10^5^) cells were seeded in Petri dishes and cultivated for 24 h in a complete cultivation medium. The sumac extract (SONNENTOR Kräuterhandels GMBH, Sprögnitz, Austria) was added to every experimental group for 24, 48, and 72 h prior to analysis.

### 4.7. Cytotoxicity Assay

The resazurin assay was used to determine cytotoxic effects of sumac extract on MCF-7, MDA-MB-231, and MCF-10A cells. The final dilutions in range of 10–350 µg/mL were prepared from DMSO extract solution. After 72 h of incubation, 10 µL of resazurin solution (Sigma) was added to each wells (final conc. 40 µM). After a minimum 1 h incubation, the fluorescence of resorufin (metabolic product) was measured at 560 nm excitation/590 nm emission filter set using the automated Cytation^TM^ 3 cell imaging multi-mode reader (Biotek, Winooski, VT, USA). The fluorescence of the control wells was taken as 100% and the results were expressed as a fold of the control. All experiments were performed in triplicate.

### 4.8. Flow Cytometry Analyses Protocol

For flow cytometric analysis, floating and adherent cells (MCF-7 and MDA-MB-231) were harvested together 24, 48, and 72 h after treatment (sumac extract final dilutions 155 or 215 µg/mL), washed in PBS, resuspended in PBS, and stained prior to analysis (see Table 7). Fluorescence was detected 15–30 min after incubation in a dark at a room temperature, using a FACSCalibur flow cytometer (Becton Dickinson, San Jose, CA, USA).

### 4.9. The Examinations of Plant Secondary Metabolites in Sumac Extract

Analytical HPLC measurements were obtained on an Agilent 1100 chromatographic system with a diode array detector 1100 series (Agilent Technologies, Santa Clara, CA, USA). Ascentis Express RP-Amide, 10 cm × 2.1 mm, particle size 2.7 µm analytical HPLC column (Sigma-Aldrich, St. Louis, MO, USA) were used, with a gradient elution of acetonitrile and 0.2% HCOOH, 0 min 10:90 (*v*/*v*), in 36th min 100% of acetonitrile. The flow rate was 0.3 mL/min, column block temperature 40 °C.

The extract was prepared from *R. coriaria* fruits powder (SONNENTOR Kräuterhandels GMBH, Sprögnitz, Austria; country of origin–Iran) by a sonication of 175 mg in 5 mL of methanol for 30 min. The extract was filtered through the 0.45 μm filter and used for analysis.

### 4.10. Statistical Analyses

All data in animal studies are expressed as mean ± SEM. The Kruskal–Wallis test, one-way analysis of variance (ANOVA), Student’s *t*-test, and Mann–Whitney test were used methods in statistical evaluations of data. The volume of tumors was calculated by the formula: *V = π* × *(S_1_)^2^* × *S_2_ / 12* (S_1_, S_2_ represent diameters of tumors; S_1_ < S_2_). Data in cell lines studies are expressed as mean ± SD and were evaluated by ANOVA followed by the Bonferroni multiple comparisons test. In the evaluation of fluorescence assay, we used ANOVA and Student–Newman–Keuls multiple comparisons test. Differences between groups were considered as significant when *p* < 0.05. GraphPad Prism, version 5.01 (GraphPad Software, La Jolla, CA, USA) was used for data analyses.

## 5. Conclusions

Isolated phytochemicals and plant foods have yet to applied in the clinical management of breast cancer. In this respect, our study evaluating the therapeutic and chemopreventive efficacy of *R. coriaria* (as a powder), using in vivo and in vitro models of breast cancer, provide novel scientific data. *R. coriaria* induced significant therapeutic efficacy in mice 4T1 adenocarcinoma allograft model and significant chemopreventive activity in chemically-induced rat mammary carcinogenesis. Oncostatic activities of sumac were accompanied by significant beneficial alterations in the histopathology of tumors found in both animal models. Mechanistic analyses of breast cancer cells in vivo and in vitro demonstrated significant proapoptotic, antiproliferative, antiangiogenic, antioxidant, anti-CSC effects and beneficial epigenetic changes after sumac treatment. All described effects of *R. coriaria* on individual cancer biomarkers found in our study refer to the activation of non-specific cell signaling pathways associated with its oncostatic activity in the therapeutic and preventive model of breast carcinoma. Systematic in-depth analysis of these pathways within preclinical and clinical oncology is needed. In addition, new treatment strategies and chemoprevention of breast cancer using whole plant foods or isolated phytochemicals require further clinical investigations, including: (a) characterizing pharmacokinetic effects; (b) effective/optimal dose; (c) determination of types of cancer cells sensitive to this type of therapy; (d) effect of treatment when used in combination with conventional therapies; (e) mode of administration; (f) achieving the sufficient levels in human plasma for beneficial effect; (g) identification of safety profile and adverse side effects during long-term administration; and (h) determining the mechanisms associated with the individual characteristics of patients.

## Figures and Tables

**Table 1:** Histopathological characteristics of 4T1 tumors in Balb/c mice after *R. coriaria* treatment.

Parameter	CONT	SUM 0.1	SUM 1
Necrosis/whole tumor area	7.65 ± 2.47	18.19 ± 5.32	7.97 ± 2.50
Mitotic activity index	38.60 ± 2.56	24.53 ± 2.86 ***	18.86 ± 1.95 ***

Data are expressed as mean ± SEM. Significant difference, *** *p* < 0.001 vs. CONT.

**Table 2:** *R. coriaria* in chemically-induced rat mammary carcinogenesis at the end of experiment.

Group	CONT	SUM 0.1	SUM 1
tumor bearing animals/all animals	25/25	22/25	20/25
tumor frequency per group ^#^	4.00 ± 0.70	2.84 ± 0.59	4.16 ± 0.81
tumor latency ^#^(days)	71.12 ± 2.79	73.36 ± 3.43	73.36 ± 3.77
tumor incidence (%)	100	88	80 *
average tumor volume ^#^ (cm^3^)	0.48 ± 0.08	0.75 ± 0.15	0.69 ± 0.13
high/low grade carcinomas ratio	42/58 (=0.724)	14/57 (0.246) **	17/87 (0.195) ***

^#^ Data are expressed as means ± SEM. Values in brackets are calculated as %-ual deviation from the 100% of non-influenced control group (with exception of latency). Significant difference, * *p* < 0.05, ** *p* <0.01, *** *p*< 0.001 vs. CONT.

**Table 3:** The cell cycle distribution in MCF-7 cells after sumac treatment.

Time (h)	24			48			72		
Treatment	CONT	DMSO	SUM	CONT	DMSO	SUM	CONT	DMSO	SUM
Sub-G_0_/G_1_	0.77 ± 0.03	1.14 ± 0.38	1.30 ± 0.20	1.23 ± 0.01	1.09 ± 0.02	1.95 ± 0.11	1.54 ± 0.13	1.42 ± 0.41	23.30 ± 3.18 **
G_1_	60.00 ± 3.43	53.35 ± 1.59 *	66.80 ± 1.96 *	68.50 ± 1.47	64.75 ± 1.84	66.55 ± 0.20	75.15 ± 0.78	70.45 ± 0.45	49.55 ± 0.12 **
S	19.45 ± 1.51	22.20 ± 1.80	18.35 ± 0.04	15.40 ± 0.16	14.25 ± 1.10	15.40 ± 1.06	11.65 ± 0.69	12.40 ± 0.73	17.65 ± 0.53
G_2_/M	19.80 ± 1.88	23.30 ± 3.02	13.55 ± 1.84 *	14.90 ± 1.63	19.90 ± 0.73	16.10 ± 1.14	11.66 ± 0.04	15.75 ± 0.12	9.50 ± 2.54

The cell cycle distribution in MCF-7cells after SUM treatment (155 µg/mL) was assessed by flow cytometry. Data are expressed as mean ± SD of three independent experiments. The significant differences between control and SUM-treated cells were signed as * *p* < 0.05, ** *p* < 0.01.

**Table 4:** The cell cycle distribution in MDA-MB-231 cells after sumac treatment.

Time (h)	24			48			72		
Treatment	CONT	DMSO	SUM	CONT	DMSO	SUM	CONT	DMSO	SUM
Sub-G_0_/G_1_	0.58 ± 0.09	0.74 ± 0.09	2.02 ± 0.16	0.69 ± 0.02	0.91 ± 0.05	4.35 ± 0.35	0.98 ± 0.02	0.99 ± 0.06	4.79 ± 0.31 *
G_1_	56.30 ± 0.49	58.20 ± 0.73	66.55 ± 1.96 *	62.30 ± 1.06	61.65 ± 1.18	64.20 ± 0.08	68.60 ± 2.04	66.75 ± 0.20	62.55 ± 0.35 *
S	19.75 ± 0.45	18.95 ± 1.51	21.65 ± 0.37	17.20 ± 0.57	16.50 ± 0.98	25.30 ± 0.01 *	11.90 ± 0.33	14.55 ± 0.53	20.45 ± 2.00 *
G_2_/M	23.35 ± 0.04	22.10 ± 0.90	9.80 ± 0.08 *	19.80 ± 0.49	20.90 ± 0.16	6.16 ± 0.13 *	18.50 ± 2.37	17.70 ± 0.41	12.20 ± 0.16 *

The cell cycle distribution in MDA-MB-231cells after SUM treatment (215 µg/mL) was assessed by flow cytometry. Data are expressed as mean ± SD of three independent experiments. The significant differences between control and SUM-treated cells were signed as * *p* < 0.05.

**Table 5:** FC analyses of apoptosis induction in MCF-7 cells after sumac (155 µg/mL) treatment.

Time (h)	24			48			72		
Treatment	CONT	DMSO	SUM	CONT	DMSO	SUM	CONT	DMSO	SUM
An^−^/PI^−^	92.05 ± 0.53	91.15 ± 0.04	88.75 ± 1.18	94.80 ± 0.24	93.60 ± 0.57	77.15 ± 0.45 **	94.75 ± 0.53	92.85 ± 0.94	56.75 ± 0.29 **
An^+^/PI^−^	3.41 ± 0.62	4.32 ± 0.88	5.10 ± 0.68	1.40 ± 0.33	2.46 ± 0.23	6.43 ± 0.35	1.96 ± 0.20	2.03 ± 0.28	4.61 ± 0.76
An^+^/PI^+^	2.41 ± 0.50	2.56 ± 0.02	3.70 ± 0.69	2.04 ± 0.14	2.39 ± 0.07	11.00 ± 0.24 *	1.97 ± 0.19	2.88 ± 0.67	29.80 ± 0.01 **
An^−^/PI^+^	2.12 ± 0.68	1.96 ± 0.89	2.47 ± 1.20	1.76 ± 0.06	1.56 ± 0.25	5.45 ± 0.54	1.35 ± 0.55	2.27 ± 0.01	8.83 ± 0.50 *

Legend: An−/PI− (lower left quadrant; non-apoptotic population), An+/PI− (lower right quadrant; early apoptotic population), An+/PI+ (upper right quadrant; late apoptotic population), An−/PI+ (upper left quadrant; necrotic population). The average of three independent experiments (±SD) is presented. Significances: control vs. SUM-treated cells * *p* < 0.05, ** *p* < 0.01.

**Table 6:** FC analyses of apoptosis induction in MCF-7 cells after SUM (215 µg/mL) treatment.

Time (h)	24			48			72		
Treatment	CONT	DMSO	SUM	CONT	DMSO	SUM	CONT	DMSO	SUM
An^−^/PI^−^	92.20 ± 1.22	92.40 ± 0.41	83.65 ± 1.21 *	90.90 ± 0.41	89.95 ± 0.86	75.30 ± 2.61 **	89.95 ± 1.67	91.65 ± 1.27	50.00 ± 3.69 **
An^+^/PI^−^	2.87 ± 0.59	2.82 ± 0.18	4.25 ± 0.53	2.51 ± 0.18	3.08 ± 0.33	6.92 ± 3.09	1.85 ± 0.62	2.20 ± 0.98	6.22 ± 1.82
An^+^/PI^+^	3.25 ± 0.36	3.01 ± 0.27	8.38 ± 1.12 *	4.06 ± 0.10	4.08 ± 0.05	12.30 ± 0.16 *	4.78 ± 0.60	3.71 ± 0.53	34.00 ± 2.10 **
An^−^/PI^+^	1.69 ± 0.31	1.78 ± 0.05	3.75 ± 0.58	2.56 ± 0.34	2.86 ± 0.48	5.46 ± 0.32	3.46 ± 0.44	2.42 ± 0.22	9.78 ± 0.92 *

Legend: An−/PI− (lower left quadrant; non-apoptotic population), An+/PI− (lower right quadrant; early apoptotic population), An+/PI+ (upper right quadrant; late apoptotic population), An−/PI+ (upper left quadrant; necrotic population). The average of three independent experiments (±SD) is presented. Significances: control vs. SUM-treated cells * *p* < 0.05, ** *p* < 0.01.

**Table 7:** List of flow cytometry analyses and staining.

Analyses	Staining Solution	Company
Cell cycle *	10% Triton X-100 0.5 mg/mL ribonuclease A 0.025 mg/mL propidium iodide–PI In 500 µL PBS	Sigma-Aldrich, Steinheim, Germany
Apoptosis	Annexin V-Alexa Fluor 647 1:100 (catalogue no. A23204)	Thermo Scientific, Rockford, IL, USA
	PI (5 mg/mL) 1:500	Sigma-Aldrich
Mitochondrial membrane potential	TMRE (tetramethylrhodamine ethyl ester per chlorate) final conc. 0.1 µM	Molecular Probes, Eugene, OR, USA
Caspase activation	Cleaved caspase-3 rabbit mAb PE conjugate 1:100 (#9978)	Cell Signaling, Danvers, MA, USA
	Cleaved caspase-7 rabbit mAb PE conjugate 1:100 (#42542)	
Proteins analysis	Cleaved PARP rabbit mAb PE conjugate 1:100 (#8978)	
	Bcl-2 mouse mAb PE conjugated 1:100 (#26295)	
	Phospho-Bcl-2 (Ser 70) rabbit mAb Alexa Fluor 488 conjugate 1:200 (#2834)	

* After harvesting, cell suspension is fixed in cold 70% ethanol and kept at −20 °C overnight.

## Data Availability

Data available in a publicly accessible repository

## Supplementary Materials

The following are available online at https://www.mdpi.com/1422-0067/22/1/183/s1.
