# Supplementary material for: Rhus coriaria L. (Sumac) Demonstrates Oncostatic Activity in the Therapeutic and Preventive Model of Breast Carcinoma
Source: Int J Mol Sci. 2020 Dec 26;22(1):183. doi: 10.3390/ijms22010183 (PMC7795985; doi:10.3390/ijms22010183)
Supplement: Supplementary file 1 [file ijms-22-00183-s001.pdf]

Product Type	Format	Reactions per Tube					Sales Order Number
PyroMark CpG Assay	Tube	200 rxn					3313126

Assay No.	Assay Name<br>GeneGlobe Cat. no.<br>Chromosomal Loaction	Gene Symbol<br>Entrez Gene ID<br>ENSEMBL Gene ID	Amplicon Length<br>Biotin Modification On<br>Sequenced Strand	Sequence to Analyze<br>Sequence to Analyze Bisulfite Converted<br>Dispensation Order	Number of CpGs	Lot No.	Date
1	Rn_Npat_02_PM<br>PM00592487<br>Chr8:56996941-56997108	Npat;Atm<br>315666;300711<br>ENSRNOG00000024934;ENSRNOG0000	167<br>Reverse Primer<br>Anti Sense	GCGACGGCCCCTGGGCGGAGTTGCCGGAGCGAGGTCGA<br>GYGAYGGTTTTTGGGYGGAGTTGTYGGAGYGAGGTYGA<br>TGTCGATCGTTGTCGATGATGTCGAGTCGTAGTCG	6	269601604	06/06/18
2	Rn_Timp3_06_PM<br>PM00574896<br>Chr7:19726892-19727057	Timp3<br>25358<br>ENSRNOG00000004303	165<br>Reverse Primer<br>Sense	CCCGGCGGGATGTGACCAAGTGGCCAAGGGTGTCTGGCGCACGAGTCCGCGGA<br>TTYGGYGGGATGTGATTAAGTGGTTAAGGGTGTTTGGYGTAYGAGTTYGYGGA<br>ATCGTCGAGTGATGATAGTAGTAGTGCTCTGTCGTATCGAGTCGTCG	6	269601606	06/06/18
3	Rn_Pitx2_08_PM<br>PM00519141<br>Chr2:226585653-226585846	Pitx2<br>54284<br>ENSRNOG00000010681	193<br>Reverse Primer<br>Sense	GGAAGCGCAGGCCGCGAGCCGGGCTCCCGCCTCCCG<br>GGAAGYGTAGGTYGYGAGTYGGGTTTTYGTTTTTYG<br>TGAGTCGTCATGTCGTCGTAGATCGTTCG	6	269601608	06/06/18
4	Rn_Pten_03_PM<br>PM00450450<br>Chr1:236769789-236769907	Pten<br>50557<br>ENSRNOG00000020723	118<br>Reverse Primer<br>Sense	GCGCTTGGGCGTCGGGACGCGGCTGCGC<br>GYGTTTGGGYGTYGGGAYGYGGTTGYGT<br>TGTCGTCTGTCGATCGATCGTCGTGTCG	6	269601610	06/06/18
5	Mm_Rassf1_02_PM<br>PM00416297<br>Chr9:107454076-107454149	Zmynd10;Rassf1<br>114602;56289<br>ENSMUSG00000010044;ENSMUSG0000	73<br>Reverse Primer<br>Sense	CGCACACGTGGTGCGA<br>YGTATAYGTGGTGYGA<br>GTCGTCATATCGATGTAGTCG	3	269601612	06/06/18
